# Passage of a Sendai Virus Recombinant in Embryonated Chicken Eggs Leads to Markedly Rapid Accumulation of U-to-C Transitions in a Limited Region of the Viral Genome

**DOI:** 10.1371/journal.pone.0049968

**Published:** 2012-11-21

**Authors:** Asuka Yoshida, Takemasa Sakaguchi, Takashi Irie

**Affiliations:** Department of Virology, Institute of Biomedical and Health Sciences, Hiroshima University, Hiroshima, Japan; University of Ottawa, Canada

## Abstract

The P gene of paramyxoviruses is unique in producing not only P but also “accessory” C and/or V proteins. Successful generation of C- or V-deficient recombinant viruses using a reverse genetics technique has been revealing their importance in viral pathogenesis as well as replication. As for Sendai virus (SeV), the C proteins, a nested set of four polypeptides C’, C, Y1, and Y2, have been shown to exert multiple functions in escaping from the host innate immunity, inhibiting virus-induced apoptosis, promoting virus assembly and budding, and regulating viral RNA synthesis. In this study, we subjected the 4C(-) recombinant lacking expression of all four C proteins to serial passages through eggs, and found the rapid emergence of a C-recovered revertant virus. Unlike the SeV strains or the recombinants reported previously or tested in this study, this was caused by an exceptionally quick accumulation of U-to-C transitions in a limited region of the 4C(-) genome causing recovery of the C protein expression. These results suggest that a lack of C proteins could lead unexpectedly to strong selective pressures, and that the C proteins might play more critical roles in SeV replication than ever reported.

## Introduction

Sendai virus (SeV; mouse parainfluenza virus type 1) is a prototype of the family *Paramyxoviridae* of the order *Mononegavirales* including some of the most important and ubiquitous disease-causing viruses of humans and animals, such as parainfluenza viruses, measles virus, mumps virus, Hendra virus, Nipah virus, human metapneumovirus, Newcastle disease virus, canine distemper virus, and rinderpest virus. SeV contains a nonsegmented, negative-strand RNA genome of 15,384 nucleotides (nt) encoding six viral structural proteins, a nucleoprotein (N), a highly phosphorylated component of the viral RNA-dependent RNA polymerase (vRdRp) complex (P), a matrix protein (M), a glycoprotein with membrane fusion activity (F) and hemagglutinin-neuraminidase activity (HN), and a large catalytic subunit of the vRdRp complex (L), tandemly in this order [1]. By recognizing the stop and reinitiation signals for transcription present at each gene boundary, the polymerase gives rise to each viral mRNA [1]. The gene expression is usually monocistronic, generating a single mRNA, which directs a single translation product. However, the P gene of paramyxoviruses is a notable exception, because it produces more than one polypeptide species by means of overlapping frames and by a process known as RNA editing of insertion of nucleotides into the transcript at a specific position during the transcription process [2]. The SeV P gene is the most diverse of those of paramyxoviruses, with at least seven polypeptides expressed from it. In addition to P protein, four C proteins (C’, C, Y1, and Y2) are translated from start codons in the +1 reading frame relative to the P open reading frame (ORF), and proteins V and W are produced from the altered P ORF with insertion of one or two G residues by RNA editing, respectively [2].

Since C protein was first found in SeV-infected cells but apparently absent in virions, they were termed nonstructural proteins [3], [4]. Subsequent studies showed that a small amount of C protein could be detected in virions, in which they appear to be associated with nucleocapsids [5], [6]. The estimated copy number of C protein in virions was as low as 40 molecules per nucleocapsid, indicating that the SeV C protein is not a major structural protein component [5].

**Figure 1:**
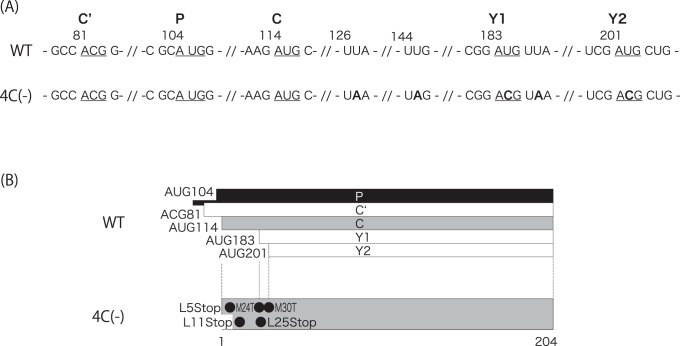
Characteristics of the recombinant 4C(-) virus used in this study. (A) Sequences around the initiation codons (underlined) of the P, C’, C, Y1 and Y2 of WT and 4C(-) are shown, and mutated codons to silence the C related proteins in the 4C(-) sequence are highlighted in bold. (B) Schematic representation of C protein of the 4C(-) recombinant. Amino acid changes for the 4C(-) mutant are indicated.

With the development of reverse genetics systems, which allow the recovery of infectious, negative-sense viruses from full-length cDNA of viral genomes, various recombinant viruses, possessing desired mutations or lacking viral proteins have been generated. A SeV recombinant 4C(-) lacking expression of all four C proteins was successfully generated using this type of system by introducing three stop codons onto upstream of the C ORF without alteration of the P polypeptide (Fig. 1; [7]). Overall titers of 4C(-) were reduced by 2-logs compared to the wild-type (WT) SeV in cultured cells, indicating that C protein is not necessary for propagation but involved in viral growth [7]. However, it was totally incapable of growing productively from the early stage of infection in mice [7].

A field pathogenic strain Ohita was rendered less pathogenic for mice through adaptation to growth in LLC-MK2 cells [8]. This attenuation was attributed to a single amino acid change from phenylalanine (F) to serine (S), at position 170 of the C protein [8]. Loss of the counteracting ability of C protein against the host innate immune system has been revealed to be responsible for the loss of pathogenicity of these viruses in mice. Indeed, one of the early-discovered functions of C protein was counteraction against IFN-signaling (JAK/STAT) by inhibiting STAT2 phosphorylation through physical interaction with STAT1 [9]–[16]. Recent studies have further demonstrated that SeV C protein also has the ability to interfere with an IFN-beta-producing pathway triggered by recognition of viral RNA by host cytoplasmic viral RNA sensors, such as RIG-I and MDA5 [17]–[20].

As an “accessory” protein, the antagonism of C protein against host innate immunity is not essential for virus replication itself, regardless of its importance to the pathogenesis in small animals. However, later studies revealed that SeV C protein also exerts other important functions at multiple stages of virus replication, such as inhibition of virus-induced apoptosis [21], regulation of the polarity of viral RNA synthesis [22]–[24] and promotion of efficient viral assembly and budding in cell cultures [7], [25]–[29]. In addition, recovery of the 4C(-) recombinant from cDNA was more difficult than that of WT or most of the other recombinant (r) SeVs, and required at least three or four passages through eggs to obtain detectable viral titers [7]. Taken together, these reports suggest that SeV C protein is not just an “accessory” protein, but play more fundamentally important roles in virus replication than expected from studies published so far.

In this paper, we examined the effect of serial passages of the 4C(-) SeV through embryonated chicken eggs, and found a quick emergence of revertant viruses caused by markedly rapid and high levels of accumulation of U-to-C transition mutations to recover C protein expression, suggesting that the lack of C protein lead unexpectedly to strong selective pressures on virus replication, and supporting the above-mentioned importance of C protein in the viral life cycle.

## Materials and Methods

### Cells, Viruses, and Antibodies

LLC-MK2 cells were maintained in Dulbecco’s minimum essential medium (DMEM; Invitrogen) supplemented with 5% fetal calf serum (FCS; PAA Laboratories, Austria) and penicillin-streptomycin (Invitrogen) at 37°C. The Polyclonal antibody (pAb) against whole virions of SeV was described previously [30]. The pAbs against the SeV P and C proteins were kindly provided by A. Kato (National Institute of Infectious Disease, Japan). SeV recombinants, 4C(-) and V(-), lacking expression of all four C proteins (Fig. 1; [7]) and V protein [31], respectively, were also provided by A. Kato. Another recombinant, F170S, possessing an F170S amino acid substitution within C protein was reported previously [17]. All of these recombinants as well as WT were recovered from cDNAs using a reverse-genetics technique on LLC-MK2 cells, and propagated in embryonated chicken eggs, as described previously [7]. Titers were determined by an immunofluorescent focus assay using LLC-MK2 cells and expressed as cell infectious units (CIU)/ml, as described previously [30].

### Serial Passage of SeV through Eggs

**Figure 2:**
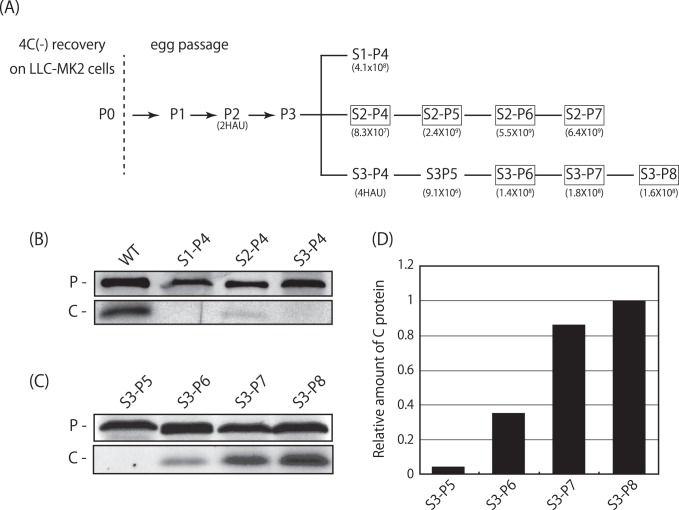
Passage history and profiles of C protein expression for the 4C(-) virus. (A) The P2 allantoic fluid stock harvested from eggs after two passages of the 4C(-) transfectant recovered from cDNA was further passed through eggs in three individual experiments. The enclosed indicate the fluid stocks with C protein expression. (B, C) LLC-MK2 cells were infected with the indicated viruses and analyzed by Western blotting using anti-P and C pAbs. (D) The amount of each C protein detected in (C) was normalized to that of P protein. The relative ratios of C to P proteins are shown as bar graphs and the ratio of S3-P8 set to 1.

We started passage of the 4C(-) virus using the P2 stock (7.2 × 10^4^ CIU/ml), kindly provided by A. Kato, which was allantoic fluid harvested from eggs after two successive passages of cells of the initial viral rescue experiment from the 4C(-) cDNA (Fig. 1B). Approximately 1 × 10^3^ CIU of the P2 stock was inoculated into the allantoic cavity of 10-day-old embryonated chicken eggs, incubated for 72 h at 34°C. Allantoic fluid was harvested from the eggs and combined as a P3 stock. After this, 100 ul of each fluid stock was passaged through eggs continually up to the eighth passage (Fig. 2A). For the other viruses, 100 ul of allantoic fluid was passaged similarly as described above. The titer of each fluid stock was determined as described above, and expression of the C protein of each stock was also examined by Western blotting as described below.

### One-step Growth Curves

LLC-MK2 cells cultured in 35 mm-diameter dishes were infected with the indicated viral fluid stocks at a multiplicity of infection (MOI) of 5. After a 1 h-incubation at 37°C, inocula were removed, and cells were washed with phosphate-buffered saline (PBS) three times, and incubated with serum-free MEM at 37°C. The culture medium was harvested at the indicated time points, and titrated as described above.

### Sequencing of Viral RNA Genome

Viral RNA was prepared from each allantoic fluid stock using a HighPure Viral Nucleic Acid kit (Roche Diagnostics) and subjected to RT-PCR using a One-step RT-PCR Kit (QIAGEN) with the primer sets of 5SeVZ1 (ACCAAACAAGAGAAAAAACATGTATGGG) +3SeVZ1066 (AGGATACAGATAAAGGGAGC), 5SeVZ995 (TATTAATAAGCTTAGAAGCC).

+3SeVZ1843 (GCGGTAAGTGTAGCCGAAGCCG), 5SeVZ1508 (GGCTGAACGGTTAGAGGAGG)+rev743NE4R (AGGACCCCAG TTACTCTTGC), and 5SeVZ2449 (GAAGCATGGAGCCTGGCAGC) +3SeVZ3572 (GGTAGGATGCCTCACCCGGG). For direct nucleotide sequencing, the RT-PCR products were sequenced twice in forward and reverse directions using a 3100 Genetic Analyzer (Applied Biosystems) with the same primer pairs used for the RT-PCR. The RT-PCR products prepared above were also subcloned into the pUC18 plasmid, and the resultant plasmid clones were subjected to DNA sequencing. At least twenty plasmid clones for each virus stock were analyzed.

Nucleotide sequences for the S2-P7, S3-P4, -P5, -P6, -P7, -P8, WT-P8, F170S-P8, and V(-)-P8 have been deposited in the GenBank database under accession numbers AB753441-9.

### Immunoblotting

LLC-MK2 cells cultured in 35mm-diameter dishes were infected with each of the 4C(-) fluid stock at an MOI of 1. After 1h at 37°C, inocula were removed, and cells were washed with PBS three times and incubated with serum-free MEM at 37°C. At 24 h post-infection (p.i.), cells were suspended in sodium dodecyl sulfate (SDS)-polyacrylamide gel electrophoresis (PAGE) sample buffer (125 mM Tris-HCl [pH 6.8], 4.6% SDS, 10% 2-mercaptoethanol, 0.005% bromophenol blue and 20% glycerol) and analyzed by SDS-PAGE (15%), followed by Western blotting with anti-SeV P or anti-SeV C pAb.

## Results

### Passage of the 4C(-) Virus Leads to a Quick Recovery of C Protein Expression

The 4C(-) recombinant virus was generated by introducing several amino acid substitutions within the C proteins without changing of the P polypeptide (Fig. 1; [7]). To silence the expression of all four C proteins, three point mutations were introduced to convert leucine residues at positions 5, 11, and 25 to termination codons, and an additional two were introduced to convert the start codons for Y1 and Y2 proteins at positions 24 and 26 to threonines (amino acid positions were assigned on the basis of those of the 204-amino acid-length C protein, as is usually done). It has been reported that recovery of the 4C(-) recombinant from the cDNA genome using a reverse genetics system is much less effective than that of most of the other rSeVs reported previously, even including the WT virus, and that detectable titers by a hemagglutination (HA) test were finally obtained after three or four successive egg passages of the transfectant [7].

The P2 allantoic fluid stock with 2 HA unit (HAU) obtained by two consecutive egg passages of the 4C(-) transfectant was further passaged through embryonated chicken eggs twice in three individual experiments, and finally the allantoic fluid stocks S1-P4, S2-P4 and S3-P4 with titers of 4.1 × 10^8^ and 8.3 × 10^7^ CIU/ml, and 4 HAU higher than that of P2 were obtained, respectively (Fig. 2A). Unexpectedly, expression of C protein was detected in LLC-MK2 cells infected with S2-P4, but not with S1-P4 and S3-P4, by Western blotting using anti-C pAb (Fig. 2B).

In terms of recovery of C protein expression of the 4C(-) virus, effects of further egg passages of two series of the P4 stocks, the C-recovered S2-P4 and the C-lacking S3-P4 up to the seventh or eighth passage, respectively, were examined (Fig. 2). Allantoic fluid stock of each passage was titrated, and inoculated into LLC-MK2 cell cultures at an MOI of 1. At 24 h p.i., expression of P and C proteins in the infected cells was examined by Western blotting using anti-P and C pAbs (Fig. 2C and 2D). In each sample, the level of P protein expression was almost equivalent (Fig. 2C). Although not detectable by the fifth passage, C protein bands were slightly appeared in the S3-P6 infected cells (Fig. 2C). The amount of C protein detected in the infected cells increased as the passages continued, indicating emergence of the revertant viruses and replacement of a major viral population occupying in the virus stocks from C-lacking to C-recovered viruses during the serial passages (Fig. 2C and D).

### Growth Characteristics of the Revertant Viruses

**Figure 3:**
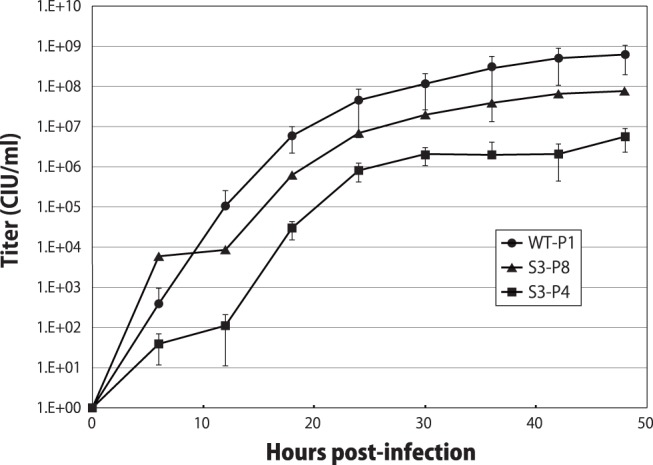
Growth kinetics of the WT and 4C(-) viruses. LLC-MK2 cells were infected with WT, S3-P4, or S3-P8 at an MOI of 5, and infectivity was determined at various time points.

To examine the effect of the recovery of C protein expression on virus replication, we compared one-step growth kinetics of the C-recovered S3-P8 stock with those of the C-lacking S3-P4 and the WT (WT-P1), which were propagated by the WT transfected cell lysate once in embryonated chicken eggs (Fig. 3). Consistent with previous reports [7],[23],[25], overall titers of S3-P4 were reduced by 2-logs compared to those of WT. Although still reduced by less than 1-log compared to those of WT, titers of S3-P8 were obviously recovered compared to those of S3-P4, indicating that the recovery of C protein expression resulted in the recovery of growth efficiency of the 4C(-) virus.

### Identification of Nucleotide Substitutions Responsible for the Recovery of C Protein

**Figure 4:**
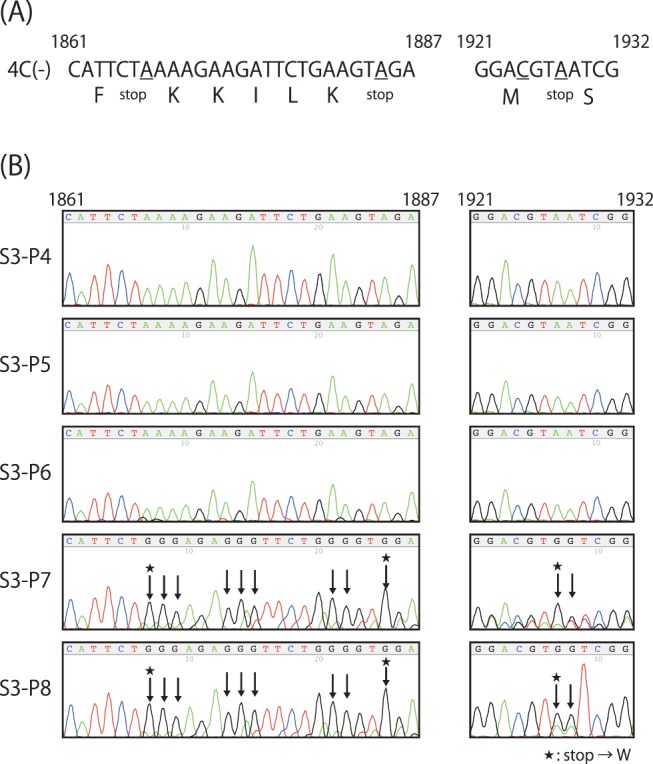
Nucleotide substitutions during serial passages of the S3 series stocks. (A) The regions of C ORF containing stop codons introduced in the 4C(-) virus are shown. (B) The electrographs of the region shown in (A) of the S3 series stocks. Appreciable A-to-G (U-to-C) transition substitutions are indicated as arrows, and among these, those causing reversions of introduced stop codons are indicated as stars.

**Figure 5:**
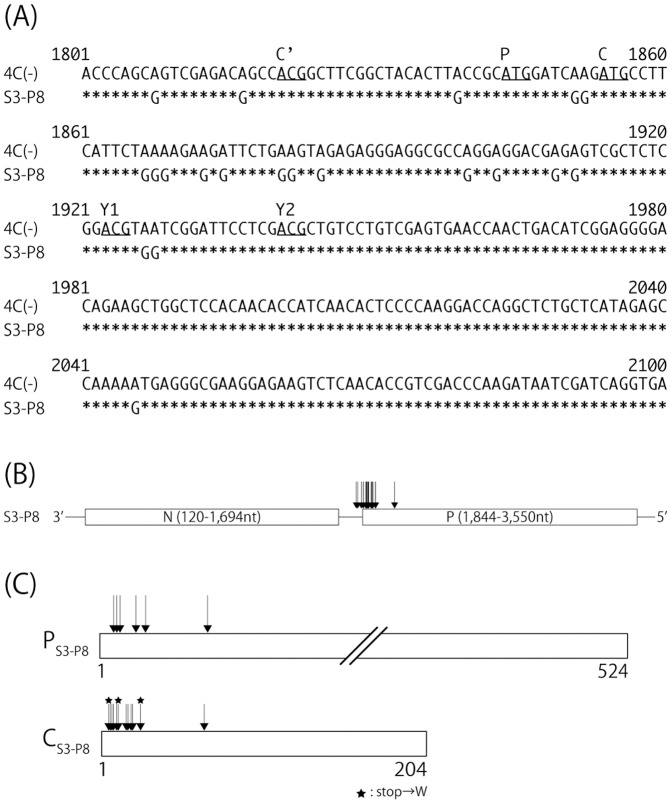
Distribution of nucleotide and amino acid substitutions observed in the S3-P8 virus stock. (A) Alignment of the 1,801 - 2,100-nt region of S3-P8 with that of the original 4C(-). Initiation codons for P, C, Y1, and Y2 proteins are underlined. Nucleotides of S3-P8 identical to that of 4C(-) are indicated as asterisks. (B) Schematic representation of a 3,700-nt region containing N and P genes of S3-P8. Arrows present positions of nucleotide substitutions. (C) Schematic representations of P and C polypeptides of S3-P8. Arrows present positions of amino acid substitutions compared to that of the original 4C(-). Stars indicate reversions of stop codons introduced into the 4C(-) genome.

To identify alterations of nucleotides in the 4C(-) genomes causing the recovery of C protein expression, we prepared bulk RNA from allantoic fluid stocks of the S3 series of fourth to eighth passages, and their nucleotide sequences of a region covering from N to P genes were compared to that of the original 4C(-) genome (Fig. 4 and 5). Unexpectedly, no nucleotide changes were found in the genome of not only S3-P4 and -P5 but also -P6, although a detectable amount of C protein was observed in the S3-P6-infected cells (Fig. 4B). However, twenty nucleotide substitutions were found within the region of the S3-P7 and -P8 samples (Fig. 4B and 5). All of these were U-to-C transition substitutions, and surprisingly, accumulated exclusively in a 1,801 to 2,100-nt region in the genomes containing a non-coding region upstream of the P gene and the beginning of the P at positions of 1,808, 1,817, 1,839, 1,851, 1,852, 1,867, 1,868, 1,869, 1,873, 1,875, 1,881, 1,882, 1,885, 1,900, 1,903, 1,909, 1,911, 1,927, 1,928, and 2,046 of the genomes (Fig. 5). These included mutations altering the three stop codons introduced in the C ORF of 4C(-) to tryptophans, responsible for the recovery of C protein expression (Fig. 5). Again, the gradual appearance of these substitutions indicates the emergence and gradual replacement of the revertant viruses to a major population in the fluid stocks during the passages. Seven and 16 of these U-to-C substitutions resulted in amino acid substitutions of P and C polypeptides, respectively (Fig. 5C). These amino acid substitutions seemed to somewhat affect replication of the revertant viruses, since one-step growth curve of the S3-P8 stock was still reduced compared to those of the WT (Fig. 2).

### Occurrence of Nucleotide Substitutions during Passages

As mentioned above, bulk nucleotide sequencing of the viruses included in the S3-P8 stock revealed that markedly quick accumulation of U-to-C transition mutations occurred within a small 100-nt region of the 4C(-) genome after only seven or eight passages through eggs. This raises possibilities that a single type of revertant virus emerged at an earlier passage became selectively dominant during further passages, or that multiple types of mutated virus with better growth than the original 4C(-) virus emerged and dominated in the fluid stocks at higher passages.

**Figure 6:**
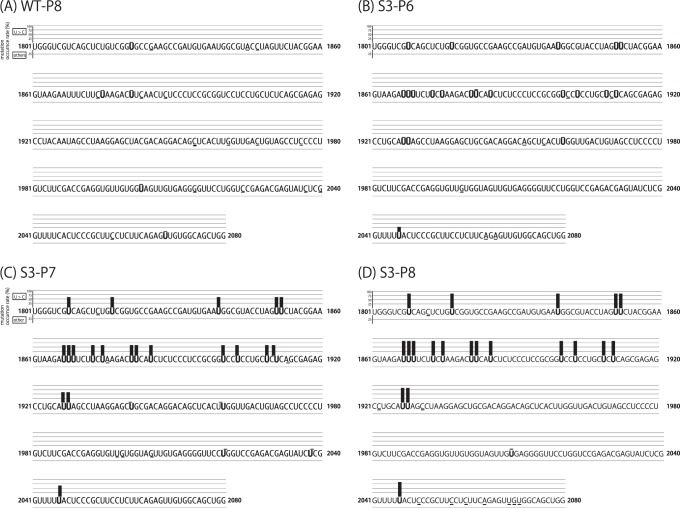
Occurrence rates of nucleotide substitutions. RT-PCR products including 1,801 - 2,100-nt regions were prepared using bulk RNAs prepared from indicated virus stocks and subcloned into pUC18 plasmids. About twenty clones of (A) WT, (B) S3-P6, (C) S3-P7 and (D) S3-P8 were sequenced and compared to those of the WT or original 4C(-). The occurrence rate of each detected nucleotide substitution is shown as bars over (for U-to-C mutations) and under (for the other types of mutations) the line of sequences of the WT or original 4C(-).

To examine these possibilities, the composition of viruses in the allantoic fluid stocks of S3-P6 to -P8 was analyzed in terms of nucleotide substitutions within the 1,801 - 2,100-nt region of their genomes (Fig. 6B–D). RT-PCR products including the region were prepared using RNA samples purified from the fluid stocks as templates, and subcloned into the pUC vector. About twenty plasmid clones of each stock were then subjected to DNA sequencing, and the nucleotide sequences were compared to those of the original 4C(-) virus. The rate of each nucleotide substitution was calculated and illustrated over and under the line of nucleotide sequence of this region as bar graphs (Fig. 6B-D). A similar experiment was performed using the WT-P8 stock, which was the allantoic fluid of the eighth egg passage of the WT virus recovered from cDNA, as a control (Fig. 6A).

**Figure 7:**
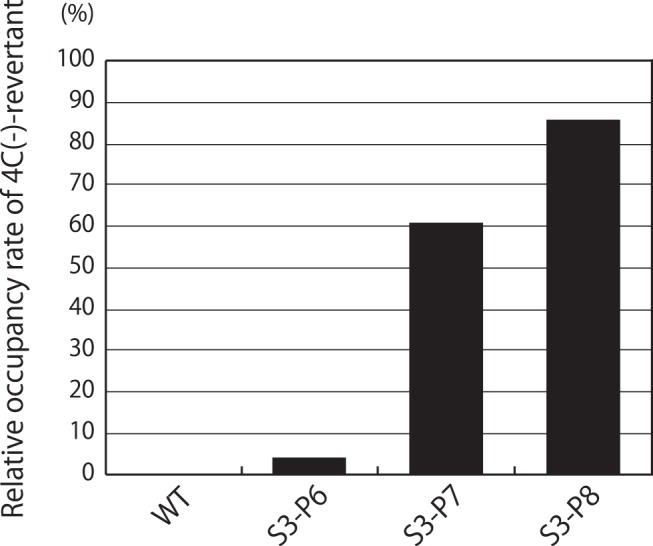
The Occupancy rate of the C-recovered mutants in each indicated virus fluid stock was shown as bar graphs.

In the WT-P8 stock, there were a variety of viruses having mutations of U-to-C as well as other types, which were observed throughout the region randomly, and the occupancy rate of each mutation against the virus population of the fluid stock was less than 5% (Fig. 6A). In contrast, in the S3-P6 stock, most (75%) of the mutations observed were U-to-C transitions, and surprisingly, the 20 U-to-C mutations were found simultaneously in the same clones occupying approximately 5% of the fluid stock (Fig. 6B and 7). This low occupancy rate might cause the undetectable C-recovering substitutions in nucleotide sequencing of the bulk RNA from S3-P6 fluid as observed in Fig. 4. As passages continued, the viruses possessing these U-to-C transitions became dominant, and finally occupied 60 and 85% of the S3-P7 and -P8 stocks, respectively (Fig. 6C, 6D, and 7). Presence of 15% of C-deficient population in the S3-P8 stock might in part contribute to its lower replication than that of the WT as observed in Fig 3. It should be noted that all of the clones possessing C-recovering reversions shared all of the 20 U-to-C mutations. Mutations other than U-to-C transitions were also observed, but seemed to occur randomly and nonsimultaneously, as in the WT-P8, and did not dominate during the passages of the S3 series stocks (Fig. 6).

### Rapid Accumulation of U-to-C Transitions of the 4C(-) Virus did not Occur in the Other SeV Recombinants

**Figure 8:**
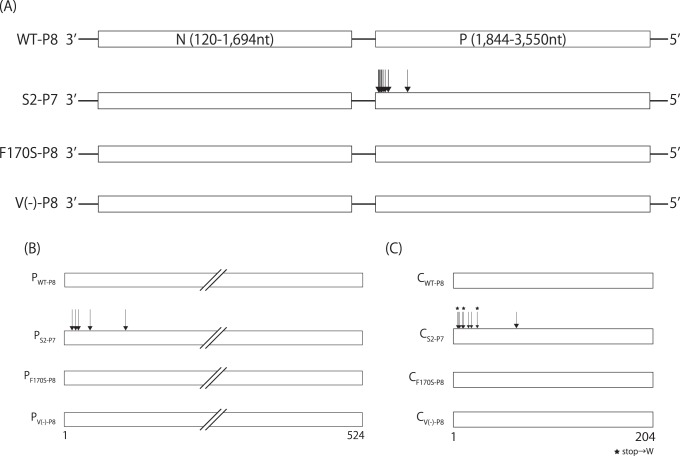
Distribution of nucleotide and amino acid substitutions observed in the WT and other recombinant virus stocks. (A) Schematic representation of regions containing N and P genes of indicated virus stocks. Arrows present positions of nucleotide substitutions. Schematic representations of (B) P and (C) C polypeptides. Arrows present positions of amino acid changes compared to that of the original 4C(-), WT, F170S or V(-). Stars indicated reversions of the stop codons introduced in the 4C(-) virus.

The rapid accumulation of U-to-C transitions in a limited region of the 4C(-) genome through egg passages implies that this region is the one allowing hypermutation or that the mutations introduced in the 4C(-) virus cause strong selective pressure for efficient virus growth. To examine these possibilities, WT as well as a C recombinant virus, F170S, and a V-knock-out virus, V(-), generated from cDNAs were passaged eight times through eggs, as the S3 series of 4C(-) was. The F170S virus possesses an F170S mutation within the C protein and the V(-) virus lacks V protein expression, and both of them have been reported to show attenuated phenotypes on pathogenesis in mice [8], [31]–[33]. In addition, the S2 series of 4C(-) virus was further passed up to the seventh passage. Bulk sequences of the passed virus stocks, WT-P8, F170S-P8, V(-)-P8, and S2-P7 were determined, and the regions spanning from N to P genes were compared in terms of the nucleotide as well as amino acid sequences (Fig. 8).

A number of U-to-C transition mutations were accumulated in the S2-P7 stock within the region where similar mutations were observed for the S3-P8 stock, and resulted in five and nine amino acid substitutions in P and C polypeptides, respectively (Fig. 8). Surprisingly, in contrast to the 4C(-) viruses, no nucleotide substitutions were found in the bulk samples of WT-P8, F170S-P8 and V(-)-P8 (Fig. 8). These results indicate that the eight passages of the SeV recombinants other than the 4C(-) are not enough for nucleotide substitutions to occur in the genomes of the bulk viruses in the stock fluids and that the quick accumulation of U-to-C hypertransitions is unique to the 4C(-) virus.

## Discussion

For the purpose of elucidating functions of the C protein, the C’/C(-) and 4C(-) recombinant viruses were first generated by Kurotani *et al.* [7]. Unlike the usual SeV recovery from cDNA using reverse-genetics, three or four blind passages of the 4C(-) transfectant were required for obtaining titers detectable by HA assay [7]. Overall growth of the 4C(-) virus was evidently lower than that of the WT in cultured cells as well as in mice [7], [23], [25].

By taking advantage of the 4C(-) virus, a variety of fundamental aspects of SeV replication and pathogenesis have been revealed. Indeed, the SeV C protein has been reported to counteract the host type-I IFN system in multiple ways [9]–[20], promote virus assembly and budding [7], [25]–[27], [29], inhibit induction of apoptosis [21], and regulate the polarity of viral RNA synthesis [22]–[24], representing the importance of C protein in virus replication and pathogenesis.

In the present study, we applied the 4C(-) recombinant to serial passages through eggs, and found quick emergence of the C-recovered revertant even after the sixth passage (Fig. 2C). As passages proceeded, titers increased in parallel with the growing occupancy rate of the revertant viruses in the fluid stocks (Fig. 2A and 7). The accumulation of U-to-C transitions in a limited region of the 4C(-) genome around upstream of the C gene occurred, and three of them caused the reversion of three stop codons introduced to knock-out C protein expression (Fig. 4 and 5). This accumulation of nucleotide substitutions occurred unexpectedly and quickly. Indeed, no substitutions were found in the bulk sequences of the fluid stocks of the WT or the other representatives tested after a similar eight passages through eggs as done for the 4C(-) virus (Fig. 8). In addition, extensive passaging of the SeV strain Hamamatsu up to fifty times was reported to lead only a few nucleotide substitutions throughout the genome [34].

Genomes of RNA viruses are error-prone during replication, because of a lack of proofreading and repair systems in the process of viral RNA synthesis. Thus, synonymous and non-synonymous nucleotide substitutions accumulate easily in RNA viruses, relative to DNA viruses. Non-synonymous substitutions (Ka) are caused by various selection pressures, while synonymous substitutions (Ks) are neutral, free from selection pressures, and accumulate as the rounds of replication increase. The ratio of Ka to Ks (Ka/Ks) represents whether an ORF has been under strong or weak selection pressures and tolerate the accumulation of mutations. Previously, we compared entire genome sequences of two SeV representatives, Hamamatsu and Z, each of distinct lineages, and calculated the Ka/Ks [35]. The strain Hamamatsu is highly virulent to rodent and not adapted to growth in eggs, while the strain Z is less virulent than Hamamatsu and adapted to growth in eggs [36], [37]. From this analysis, it was revealed that the C-terminal two thirds of the C protein (C2; Ka/Ks  = 0.008) was much less flexible than the remaining N-terminal one-third of the C protein (C1; Ka/Ks  = 0.602), in terms of allowance of non-synonymous changes [2], [35]. In addition, the region in the P ORF corresponding to C1 (PC1; Ka/Ks  = 0.211) had similar flexibility to C1, although that corresponding to the C2 (PC2; Ka/Ks  = 2.283) was much more flexible than the other regions [2], [35], which is consistent with the mutational flexibility of P proteins reported for a number of mononegaviruses [38]. These results suggest that the region containing C1 and PC1 might be more tolerable to amino acid substitutions than the remaining region of C protein, C2. Indeed, nucleotide substitutions altering amino acids of P and C proteins were found exclusively within this region in the C-recovered samples (Fig. 5 and 8).

The mechanism by which the nucleotide substitutions accumulated quickly in a limited region of the 4C(-) genome has not been elucidated yet. However, incidental U-to-C transitions as well as other types of mutations were found randomly in the 4C(-) genome as observed in the WT, and a single type of the 4C(-) revertant became dominant in the population of the fluid stocks after several egg passages (Fig. 6 and 7). These observations suggest that numerous types of viruses possessing incidental U-to C transitions emerged during passages, and among these incidentally emerged viruses, the most dominant one with recovered expression of C protein survived under strong selective pressures from a lack of C protein functions.

Such biased transition hypermutation as observed for the 4C(-) SeV has been well characterized for measles virus (MeV). A host double-stranded RNA-specific adenosine deaminase ADAR1 is induced by MeV infection, and U-to-C transition substitutions are introduced exclusively within the M gene, resulting in emergence of defective viruses, which cause subacute sclerosing panencephalitis (SSPE) [39]–[41]. For some other viruses, ADAR1 has been reported to induce mutations into restricted regions of the viral genomes and to enhance virus replication [42]–[44]. ADAR1 is more likely to be involved in the markedly rapid hypermutation in the limited region of 4C(-) genome during egg passages, rather than random mutagenesis during unfaithful viral RNA synthesis is, because the C-recovering reversion requires at least three U-to-C transitions simultaneously at three specific positions. However, the hypermutated region is unlikely to be directing mutagenesis induced by ADAR1, since accumulated mutations were not observed in this region of all of the viruses tested other than the 4C(-) (Fig. 8). Conceivably, the presence or absence of C protein affects the activity of ADAR1 or the acceptability of ADAR1-induced mutagenesis in a specific region of SeV genome.

Altogether, the 4C(-) virus might be more deficient in replication than believed, because it seems to be exposed to severer selective pressures than expected, which might be why three or more blind passages through eggs were required for obtaining detectable titers of this virus. This suggests the critical importance of C protein in SeV replication, and that even in the experiments using the “C-lacking” 4C(-) virus performed previously: unidentified mutations in the genomes during egg passages might somewhat complement the functions, and obfuscate the “real” importance of C protein. Detailed characterization of the 4C(-) clones isolated from the fluid stocks would be interesting and important, and give a deep understanding of functions of C protein as well as paramyxovirus replication.
